# Young and adult patients with gaming disorder: Psychiatric co-morbidities and progression of problematic gaming

**DOI:** 10.3389/fpsyt.2024.1423785

**Published:** 2024-12-10

**Authors:** Annika Hofstedt, Anna Söderpalm Gordh

**Affiliations:** ^1^ Sahlgrenska Academy, Institution for Neuroscience and Physiology, Section for Psychiatry and Neurochemistry, University of Gothenburg, Gothenburg, Sweden; ^2^ Department of Addiction and Dependency, Clinic for Gambling Disorder and Screen Health, Sahlgrenska University Hospital, Gothenburg, Sweden

**Keywords:** gaming disorder (GD), adolescents and young adults (AYAs), adults (MeSH), age, progression, psychiatric co-morbidity

## Abstract

**Background:**

Previous research suggests age-dependent differences in the progression of addiction, and evidence is accumulating, showing that an early initiation of gaming increases the risk for addiction. With the recent introduction of gaming disorder (GD) as a psychiatric diagnosis, there is a need to extend the knowledge of the clinical characteristics of patients seeking treatment for GD of all age groups. Compared to adolescents and young adults, less is known about treatment-seeking adults. This study aimed to investigate whether there are clinically relevant age-dependent differences among patients seeking treatment for GD.

**Method:**

Participants were recruited among patients seeking treatment and fulfilling diagnosis for GD at an outpatient clinic specialized in the treatment of GD. During the study period, 142 patients went through assessment for GD at the clinic, 37 did not fulfill the diagnosis for GD, and 36 declined participation, leaving a sample of 69 patients (age range = 15–56) for analysis. The sample (men, *n* = 66; women, *n* = 3) was divided in two age groups: adolescents and young adults (25 years or younger) and adults (26 years or older). Gaming-related data and information about psychiatric co-morbidity was collected through structured clinical interviews and questionnaires.

**Results:**

The adolescents and young adults (AYAs) reported a more rapid progression into problematic gaming than the adult group. The younger group developed problematic gaming four years faster than the adults. We also observed comparable clinical profiles in both groups. Both age groups had similar levels of GD as well as symptoms of psychiatric co-morbidities including possible attention deficit hyperactivity disorder (ADHD), autism spectrum disorders (ASD) and problematic gambling. We also noticed that half of our study population consisted of adults.

**Conclusion:**

With the increasing prevalence of gaming in all age groups, it is unknown how the occurrence of GD will develop in different stages of life. We conclude that the adolescents and young adults had almost double as fast progression to problematic gaming than the adult group, highlighting the need for preventive strategies. The similarity in clinical profiles indicates that treatments with the same type of interventions could be offered to both age groups.

## Highlights

- Problematic gaming exists in all age groups, but adults are often excluded in treatment studies. Therefore, we wanted to compare young and adult patients with gaming disorder.
- Half of our patients were adults.
- The younger patients were 14 years old and the adults were 21 years old when they first developed problems with gaming.
- Many of our patients also had symptoms of ADHD or autism.
- We suggest that the same type of treatment could be useful for both adolescents and adults with gaming disorder.

## Introduction

Gaming has rapidly increased during the last decades. Technological advances have made it possible to design more complex games offered through a variety of platforms, and online gaming is now available around the clock. Gaming is now a widespread popular activity in both younger and older age groups (1), and it is predicted that more than one-third of the global population will be videogame players in the end of 2024 (2). For most people, gaming is a pleasurable pastime, but for some gaming gradually comes to dominate daily activities, causing significant impairment that develops into a gaming disorder (GD).

The phenomenon of being addicted to videogames has been reported as early as in the 1980s, although a large increase of studies was observed parallel with the introduction of online gaming in the 2000s (3). In 2019, GD was included by the World Health Organization (WHO) as a new diagnosis in the International Classification of Diseases (ICD-11) under the section for substance use and addictive behaviors (4, 5). Gaming disorder is defined by impaired control over gaming, increasing the priority given to gaming and continuation of gaming despite the occurrence of negative consequences. A similar diagnostic construct named Internet gaming disorder was included among “Conditions for Further Studies” in the Statistical Manual of Mental Disorders (DSM-5) (6). In DSM-5, it is described that individuals with GD often devote at least 8–10 h per day and a minimum of 30 h per week to gaming. The amount of time spent gaming is, however, not a valid discriminator between engaged gamers and those with GD (5, 7). Even though the time spent on gaming is not a diagnostic criterion, it has been found to be positively associated with GD. In a cross-sectional study with adolescents, it was found that those fulfilling GD typically spent an average of 5 h per day gaming, whereas the non-addicted gamers generally spent around 3 h (8). Furthermore, in a sample consisting of both teenagers and adults, the average time spent gaming among those at risk for GD was 42 h per week, while those not at risk played on average 24–26 h per week (9).

Recent meta-analyses indicate global prevalence rates for GD of 1.96% and 1.39%, respectively, when only including studies with representative samples (10, 11). However, studies show global differences, with especially high prevalence in Asian countries (10, 11). There are also indications that the prevalence of GD is rising, especially among women, although analyses show that part of this rise might be attributable to changes in ways to measure GD (11). There are further considerable differences in the prevalence rates between different demographic groups. Gaming disorder is 2.5 times as common among men than women (11), and about three times as common among children, adolescents, and young adults compared to adults (10). There is also an association between the prevalence rates and types of games being played, with genres such as massively multiplayer online-playing games (MMORPGs), first-person shooter (FPS), and multiplayer online battle arena (MOBA) being more associated with GD (12) as well as online games more than offline games (13).

Although there is a scarcity of longitudinal studies, research suggests that starting gaming at a younger age is a risk factor for the development of GD (14) and that early exposure can contribute to more severe levels of GD (15, 16). An association between younger age of initiation and development of addiction has also been found in other behavioral addictions. Two large cross-sectional national surveys found an increased risk for gambling disorder when gambling is started at an early age (17, 18). Similar findings have also been reported regarding the early use of the Internet (19, 20). This mirrors earlier research on substance use disorders. Research has shown that early experiences of drugs—for example, alcohol—are associated with a higher risk for dependence and related problems during adult life (21). Individuals with an early exposure to drugs have also been found to progress faster to addiction than those with a later onset (22, 23). It has also been described that those who begin drinking at an early age (around 12 years of age) are more heavily genetically predisposed to alcohol use disorder (AUD) compared to those who start drinking at an older age (24). Interestingly, in a longitudinal twin study, time spent gaming was similarly found to be dependent on genetics with a heritability of 19%–63%, with an increased genetic contribution at older ages for boys, but not for girls (25).

Comparably to other addictions, research has shown that psychiatric co-morbidity is common among those with GD. Closely related to GD is depression and anxiety (13, 26–28). Meta-analyses have also shown positive correlations between ADHD and GD (29) and between ASD and GD (30). Studies also indicate a possible relationship between excessive gaming and psychosis in young people (for a summary see 31) and that overexposure to videogames could trigger a psychotic onset or worsen a pre-existing psychosis (32, 33). Gaming disorder also appear together with the use of a variety of substances in the adult population [see the review by Burleigh et al. (34)]. Problematic gaming has further been shown to co-occur with cigarette smoking, nicotine use, alcohol, caffeine, and cannabis use (35–39) as well as more frequent substance use and polysubstance use (40). Interestingly, in contrast to the findings mentioned above, a heavy investment in gaming has also been found to be associated with lower alcohol use in adults (41). Studies further suggest that there are age differences in psychiatric co-morbidity, with older individuals with GD being more heavily burdened with co-morbidity (16, 26).

Other individual factors have also been linked to GD. For example, associations with personality traits have been reported, in particular, high neuroticism and low conscientiousness (42). Difficulties in identifying one’s emotions, i.e., alexithymia, has been linked to the problematic use of the Internet in general (43) and more specifically to GD (44). Furthermore, having low self-esteem has as well been linked to GD (15). These individual factors may contribute to an increased risk of GD, but they may also be reinforced by extensive gaming in a bidirectional relationship. In addition, it has been found that specific motives for gaming are more strongly associated with GD than others, with escapism repeatedly being identified as having the strongest relationship (45–47). Similarly, spending more time online and also more time playing videogames has been described to increase during pandemic-related stress, possibly as a coping strategy (48).

Research about GD has mostly focused on younger populations. Adults are often omitted both in studies with clinical samples (49, 50) and in studies about GD and psychiatric co-morbidity in the general population (26, 27). Moreover, treatment studies have often excluded adults. In a brief oversight of the literature on treatments for GD, we found three reviews (49–51), one meta-analysis (52), and one mix of a review and a meta-analysis (53). These included almost 100 unique papers, and of these only 1/4 included adults (>25 years) as participants. Even though GD is more prevalent among the younger age groups (11), indications of age-dependent differences in co-morbidity (16, 26) as well as the increased prevalence of gaming in all age groups, highlight the importance of including individuals of all ages when conducting research about GD.

This study aimed to expand the knowledge about patients seeking treatment for GD and specifically to investigate possible differences in clinical profiles between adolescents and young adults (AYA) and adult patients. As most clinical studies focus on younger patients, this adds valuable knowledge for designing treatment options suitable for both AYAs and adults with GD. In line with previous research on alcohol, we hypothesize that an early debut of gaming leads to a faster progression of GD. We also hypothesize that the adults will have more psychiatric co-morbidity than the AYAs.

## Materials and methods

### Study design

This study was an observational cross-sectional study. The sampling method was a non-probability convenience sample, as patients were able to decline participation. The data was collected between February 2020 and March 2024, and the participants were continuously recruited as they sought treatment at the study site. The study sample was divided into two groups, based on age at seeking treatment, after the data had been collected. The information used in the study was obtained through semi-structured interviews and standardized questionnaires. The Swedish Ethical Review Authority, dnr 764-18, had approved the study, and it was conducted according to the 1964 Declaration of Helsinki.

### Participants and procedure

The participants (*n* = 69) were recruited from the Clinic for Gambling disorder and Screen Health, Department of Addiction and Dependency, at Sahlgrenska university hospital in Gothenburg, Sweden. The clinic is the largest public health outpatient facility offering treatment for gaming disorder in Sweden and welcomes patients with GD from the year they turn 16, with no upper age limit set. The treatment offered is based on cognitive behavioral therapy. Patients are referred to the clinic either by self-referral or by referral from a physician or other healthcare professionals. To be included in the study patients had to fulfill diagnostic criteria for GD according to a diagnostic interview. No specific exclusion criteria were used in the study.

At their first visit to the clinic, the patients were informed about the study and approved participation. The participants were assessed with a semi-structured anamnestic interview, and the fulfillment of GD diagnosis was assessed through a semi-structured diagnostic interview for GD. The diagnostic assessments were made by a clinical psychologist, a social worker, or a nurse and were then validated at a treatment conference where a clinical psychologist made the final decision about diagnoses. In addition, sociodemographic data was collected, and several questionnaires were administered to measure the severity of GD and symptoms of psychiatric co-morbidity. In addition, we assessed other clinically relevant factors such as progression into problematic gaming, reasons for gaming, and preferred gaming genres. During the study period, 142 patients were offered to participate in the study. Out of the 142 participants, 37 were excluded, as they did not fulfill the diagnostic criteria for GD. Furthermore, 36 participants declined to participate by not giving their consent. This left a total of *n* = 69 participants for analysis.

### Measures

#### Anamnestic interview

The anamnestic interview was created on site purposely for this clinical setting, and questions were asked about tobacco use, drug use, and other psychiatric diagnoses besides gaming. We also collected information related to gaming about age of gaming debut, duration of gaming problems, debut of gaming problems, days of gaming per week, hours of gaming per week, reasons for gaming, and preferred game genres.

#### Diagnostic interview

The diagnostic interview was based on the diagnostic criteria for Internet gaming disorder from the DSM-5 (6). The interview was adapted from a version developed by Vadlin et al. (54). It consists of structured questions in relation to each diagnostic criterion to aid in the decision on whether the diagnosis is fulfilled. According to the instructions in the DSM-5, the disorder is present if at least five of the nine criteria are fulfilled during the last 12 months.

#### Self-report questionnaires

Gaming addiction identification test (GAIT) is a screening tool for GD developed and validated in a Swedish population. GAIT contains 17 questions about gaming and covers all the diagnostic criteria for Internet gaming disorder from the DSM-5 with a very good internal consistency (Cronbach’s alpha = 0.95). A version of the test covering gaming in the past 30 days was used. The test questions are about digital games not only on computer but also games on mobiles or TV (54). The suggested cutoff for GD is to fulfill at least five questions as “completely agree.”

Patient Health Questionnaire (PHQ-9) contains nine questions screening for symptoms of depression in the last 2 weeks. The questionnaire is developed according to the diagnostic criteria in DSM-IV, and the scores assess the severity of depressive symptoms. The total score corresponds to the level of severity and is classified as none (0–4), mild (5–9), moderate (10–14), moderately severe (15–19), or severe (20–27) depression. The PHQ-9 has a high validity in detecting the severity of depression (Cronbach’s alpha = 0.89) (55).

Generalised Anxiety Disorder Assessment (GAD-7) was developed to measure the symptoms of anxiety. The GAD-7 consists of seven questions screening for symptoms of anxiety in the last 2 weeks. The total score is 21, indicating minimal (0–4), mild (5–9), moderate (10–14), and severe (15–21) levels of anxiety (Cronbach’s alpha = 0.92) (56).

*NODS-PERC* is a short screening instrument for pathological and problem gambling that consists of four yes or no questions measuring gambling problems the last 12 months. One yes or more indicates possible gambling problems (sensitivity of .997; specificity = .394; PPV = .885; NPV = .963; and diagnostic efficiency = .891) (57).

Alcohol Use Disorders Identification Test (AUDIT) is a screening instrument for alcohol-related problems (Cronbach’s alpha = 0.82). The test consists of 10 items allocated in three areas: alcohol consumption, symptoms of dependence, and negative consequences of alcohol consumption. The cutoff score of 6 for women and 8 for men, respectively, indicates hazardous or harmful drinking. The maximum score is 40 (58).

Drug Use Disorders Identification Test (DUDIT) is a screening test for use of illicit drugs and drug-related consequences. It consists of 10 items with a maximum score of 40. The questionnaire is categorized in three drug use areas: drug use, drug dependence symptoms, and negative consequences of the drug. Scores of 1 or more for women and 3 or more for men indicate problematic drug use (Cronbach’s alpha = 0.80) (59).

Adult ADHD Self-Report Scale-V1.1 (ASRS-V1.1) Screener is a screening instrument designed to identify adult individuals with symptoms of ADHD. The test consists of six questions describing different symptoms related to ADHD with a five-point response scale ranging from “never” to “very often”. Each question is scored dichotomous, with each symptom considered prevalent if responding “sometimes”, “often”, or “very often” to the first three questions and “often” or “very often” to the remaining three questions. Four or more positive symptoms indicate possible ADHD. It is not a diagnostic tool but is meant to be used to identify individuals in need of a more thorough assessment for ADHD. The sensitivity is 68.7%, and the specificity is 99.5% (60). The ASRS screener was originally developed for and validated in adult samples of ages 18 years and above, but studies have since shown it to be a reliable and valid measure also in samples from adolescents (61).

Ritvo Autism and Asperger Diagnostic Scale Screen (RAADS-14 Screen) is a screening instrument for ASD in an adult population and is based on an original 80-item questionnaire. The test consists of 14 questions and has a four-item scale that ranges from “never true” to “true now and when I was young”. A score of 14 or above is judged to be the optimal cutoff to identify possible ASD in a psychiatric outpatient sample (Cronbach’s alpha = 0.80) (62).

The demographic data questionnaire assesses a number of demographic characteristics from the participants including age, gender, educational level, occupational status, living situation, and current occupation. This demographic questionnaire was specifically created for this study.

### Data analysis

The data analysis was performed using IBM SPSS Statistics version 28. All the hypotheses were tested with a significance level of *α* = 0.05. The participants were divided in two groups: a younger group consisting of AYAs and an adult group. The age span for AYA was defined as ages up to 25 (63–65), and the adult group consisted of those who were 26 years of age or older.

The information received in the anamnestic interview about the participants’ reasons for gaming was clustered in four categories (escape and coping, habit, improve ranking, and social), and the types of games that the participants preferred were clustered into six genres (MMORPG, FPS, MOBA, sport games, mobile games, and other). The participants were allowed to answer with several reasons and game genres, and therefore the total frequency exceeds 100%.

A large proportion of the answers about hours gaming on a typical day and number of gaming days per week were on the highest possible option in the GAIT questionnaire (10 h a day and 4 days per week, respectively), indicating a possible ceiling effect. These variables were thus analyzed as dichotomous variables. For gaming hours on a typical day, we made three separate calculations, dividing the participants in groups depending on time spent gaming per day (up to 5 h, 6 to 7 h, and 8 h or more). For gaming days per week, two groups were constituted of those indicating the highest option, 4 days or more, and the ones indicating less than 4 days per week.

Differences between AYA and adults in categorical variables (time spent gaming per day and week, ASRS, RAADS-14, NODS-PERC, tobacco use, family history of addiction, education, occupational status, living situation, reasons for gaming, and gaming genres) were tested with Fisher’s exact test as this gives more exact statistics for cross-tabulations with 2 × 2 cells and works in larger cross-tabulations where the expected count in >20% of the cells is less than five, which was the case for all of our larger cross-tabulations (66).

Histograms of the continuous variables age, age of gaming onset, debut of problem gaming, years to develop gaming problems, duration of problem, number of DSM-5 criteria, GAIT, PHQ-9, GAD-7, AUDIT, and DUDIT were examined to analyze skewness. The distributions were judged to be approximately normally distributed, and therefore we continued the analyses with parametric statistics. A two-tailed *t*-test was used for the continuous variable *age* to test for a difference between the groups. In the remaining tests, AYA and adults were used as the dependent variables. Possible ADHD was, on a theoretical basis, judged to be a possible confounder for both the gaming variables and the measures of other types of psychiatric symptoms (29, 67) and was included as a covariate. The participants were coded as having possible ADHD if they screened positively for ADHD on the ASRS scale. To analyze possible differences between the groups and control for confounders, analysis of covariance (ANCOVA) was used. As the study was exploratory in nature, no correction was made due to multiple testing, and no power calculation was carried out to determine the sample size.

For clinical reasons, the battery of questionnaires was changed during the collection of data, and therefore *n* = 24 is missing on DUDIT. The number of missing data points was between 0% and 17% per variable, on average 6.2%, not counting the missing information regarding DUDIT.

We also calculated Cohen’s *d* for all continuous variables and odds ratios for the dichotomous variables. Cohen’s *d* was calculated with estimated marginal means and original standard deviations. The odds ratios are reported as the odds of the event among AYAs divided by the odds among adults.

## Results

### Sociodemographic characteristics

Sociodemographic characteristics of the total group of participants (*n* = 69) as well as for the young (*n* = 35) and the old (*n* = 34) groups together with test statistics and *p*-values are presented in **Table 1**. The mean age of the AYA group was 21.2 (SD = 3.1; age range 15–25), and the adult group had a mean age of 33.5 years (SD = 7.9; age range 26–56). Fisher’s exact test (*p* = .001) showed a significant association between age group and education level. The AYAs more frequently had less than high school education, while the adults more often had university level education. There was also a difference between the groups regarding occupational status according to Fisher’s exact test (*p* = .001), with the adults more often working and the AYAs more often studying. According to Fisher’s exact test, a significant difference was found in living situation (*p* = .001). The adults lived more often alone or with a partner compared to the AYAs who more often lived with relatives or friends.

**Table 1:** Demographic information.

Variables	Total (n=69)	Younger/AYA (n=35)	Older/Adult (n=34)	*p*-value	Effect size (Odds ratio)
**Age** M (SD)	27.3 (8.6)	21.2 (3.1)	33.5 (7.9)		
**Age range**	15-56	15-25	26-56		
**Gender** (% male)	95.7	97.1	94.1	–	–
**Education** %*				<0.001	
Less than high school	28.8	47.1	9.4		OR = 8.59
High School	40.9	41.2	40.6		OR = 1.02
Occupational training	12.1	5.9	18.8		OR = 0.27
University	18.2	5.9	31.3		OR = 0.14
**Occupational status** %*				<0.001	
Working	27.5	11.4	44.1		OR = 0.16
Studying	34.8	57.1	11.8		OR = 10.0
Sick-leave	11.6	11.4	11.8		OR = 0.97
Unemployed	21.7	17.1	26.5		OR = 0.57
Other	4.3	2.9	5.9		OR = 0.47
**Living situation** %*				<0.001	
Alone	29.4	20.0	39.4		OR = 0.38
With partner	10.3	0.0	21.2		–
With partner and children	13.2	0.0	27.3		–
Single parent	1.5	0.0	3.0		–
With relatives/friends	45.6	80.0	9.7		OR = 40.0

Difference in age was calculated with a t-test.

Data is presented as means and standard deviations *M* (*SD*), in range and in percent (%).

Education, occupation status and living situation were calculated with Fishers exact test.

Effect size is reported as odds ratios for the categorical variables.

OR, Odds ratios are reported for AYAs to adults.

*Statistically significant.

### Gaming-related measures

The results from the ANCOVAs assessing the differences in gaming related measures between the younger and the older group, controlling for the confounder ADHD, *F*-values, and *p*-values, are reported in **Table 2**.

**Table 2:** Gaming related measures.

Variables		Total (n=69)	Younger/AYA (n=35)	Older/Adult (n=34)	*p*-value	Effect size (Cohen’s *d* / Odds ratio)
**Age of gaming debut**	Unadjusted	8.4 (6.3)	6.7 (2.7)	9.9 (8.0)		
	Adjusted	8.6 (6.8)	6.9 (2.8)	10.3 (8.9)	0.07	*d* = 0.58
**Debut of problem gaming** (age in years)*	Unadjusted	17.4 (7.7)	13.9 (3.7)	21.0 (9.1)		
	Adjusted	17.7 (8.1)	14.0 (3.9)	21.8 (9.5)	<0.001	*d* = 1.14
Years to develop problems*	Unadjusted	9.1 (6.0)	6.9 (3.8)	11.1 (6.9)		
	Adjusted	9.2 (6.3)	6.8 (4.0)	11.6 (7.2)	0.005	*d* = 0.88
**Duration of problem** (years) *	Unadjusted	9.9 (6.1)	7.3 (4.8)	12.6 (6.3)		
	Adjusted	9.8 (5.9)	7.5 (4.8)	12.4 (6.0)	0.001	*d* = 0.90
**GAIT** (Gaming addiction identification test)	Unadjusted	41.4 (8.7)	40.0 (8.4)	43.0 (8.9)		
	Adjusted	41.5 (9.0)	39.9 (8.7)	43.3 (9.1)	0.18	*d* = 0.38
Number of DSM-criteria*	Unadjusted	7.0 (1.3)	6.7 (1.3)	7.3 (1.2)		
	Adjusted	7.0 (1.3)	6.7 (1.3)	7.4 (1.2)	0.018	*d* = 0.66
**Gaming days** at least 4 days/week (%)		84.7	82.1	87.1	0.72	OR = 0.68
**Gaming time** at least 8 hours/day (%)		44.1	50.0	38.7	0.44	OR = 1.58
**Gaming time** 6-7 hours/day (%)		27.1	25.0	29.0	0.78	OR = 0.81
**Gaming time** up to 5 hours/day (%)		28.8	25.0	32.3	0.58	OR = 0.70
**Reasons for gaming %**						
Escape/coping		80.9	76.5	85.3	0.54	OR = 0.56
Habit		35.8	39.4	32.4	0.62	OR = 1.36
Improve ranking		45.6	44.1	47.1	1.0	OR = 0.89
Social		44.9	48.6	41.2	0.63	OR = 1.35
**Gaming genre %**						
MMORPG		51.5	51.4	51.5	1.0	OR = 1.0
FPS*		39.7	57.1	21.2	0.003	OR = 4.95
MOBA		20.6	25.7	15.2	0.37	OR = 1.94
Sport games		4.4	0.0	9.1	0.11	–
Mobile games*		11.8	2.9	21.2	0.025	OR = 0.11
Other		1.5	0.0	3.0	0.49	–

Data is presented as means and standard deviations *M* (*SD*), in percent (%).

Gaming days, gaming time (8, 6-7, 5 hr/day) gaming reasons and gaming genre were calculated with Fisher’s exact test. Age of gaming debut, debut of problem gaming, time to develop problem, duration, GAIT, number of DSM-criteria was calculated with ANCOVA and presented in the table with adjusted and unadjusted means and standard deviations *M* (*SD).*

Effect size is reported as odds ratios for categorical variables and Cohen’s *d* for continuous variables. OR, Odds ratios are reported for AYAs to adults.

*Statistically significant.

**Figure 1:**
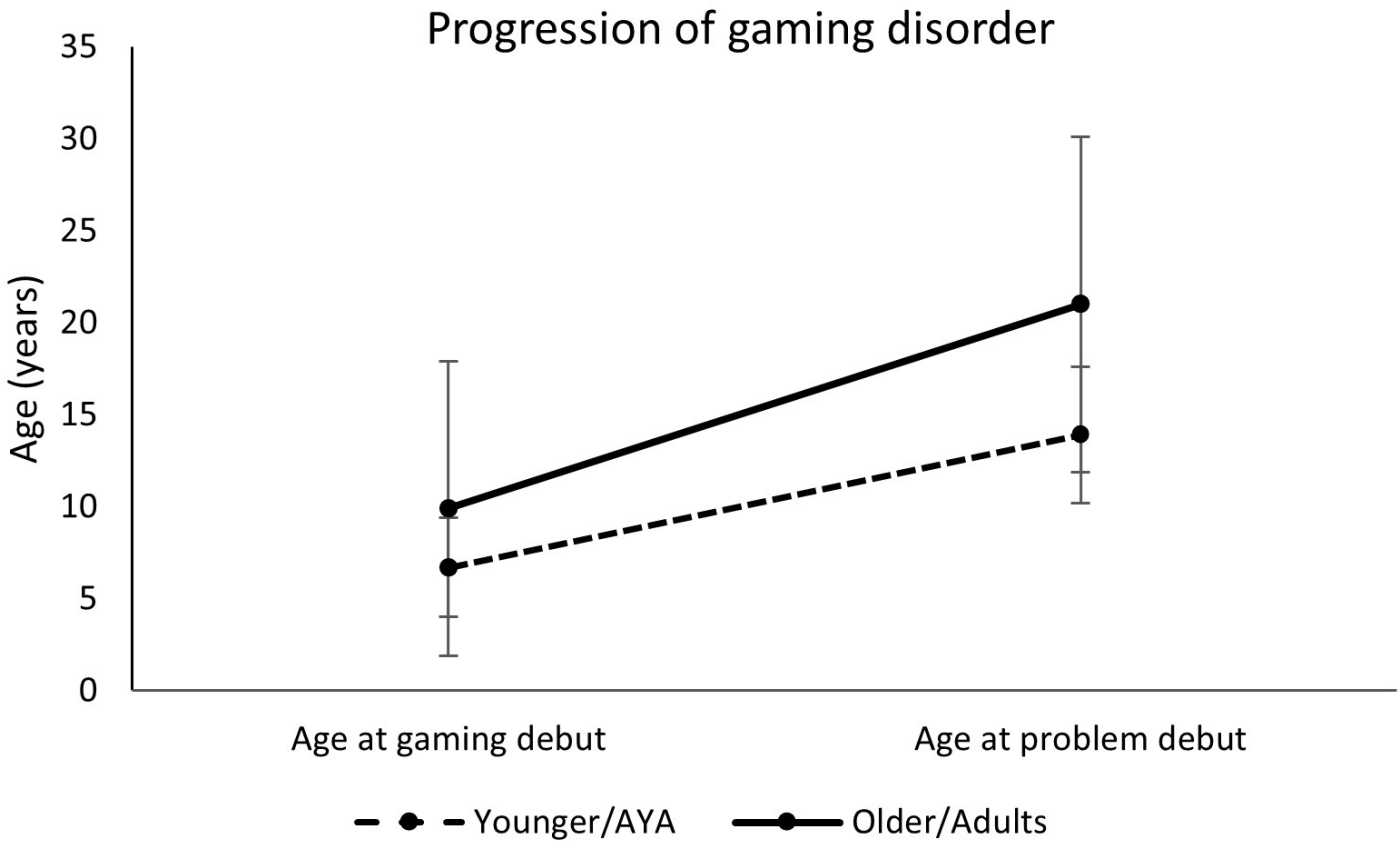
Progression of gaming disorder in AYA and adults, shown as the time between debut age of gaming and debut of problem gaming.

First, in ANCOVA, when controlling for ADHD, the groups did not differ in age of gaming debut F(1,53) = 3.4, *p* = .07 and ADHD F(1,53) = 0.06, *p* = .80. However, if not controlling for ADHD, the groups differed in age of gaming debut F(1,64) = 4.27, *p* =.04 (6.7 vs 9.9 years). We believe that this could be due to a power problem because of the lower *n* when ADHD was considered in the model. Second, in ANCOVA, when controlling for ADHD, the groups significantly differed in debut age of problem gaming [F(1,57) = 17.1, *p* <. 001; ADHD F(1,57) = 0.12, *p* = .73], showing that AYAs were on average 14 years old and the adults were almost 22 years old when they started having problems with gaming. Third, ANCOVA revealed, when controlling for ADHD, that the groups also differed in time to develop gaming problems. It was demonstrated that AYAs developed problems about 7 years after gaming debut and the adults after 11 years [F(1,53) = 8.8, *p* = .005; ADHD F(1,53) = 0.12, *p* = .74]. Additionally, in ANCOVA, when controlling for ADHD, the groups also differed in duration of problems. It was found that AYAs had a shorter duration of problems compared to the older group (on avarage 7.3 versus 12.6 years) [F(1,57) = 12.0, *p* = .001; ADHD F(1,57) = 0.09, *p* = .77]. Debut age in gaming, debut of problem gaming, and the time to develop problems are shown in **Figure 1**.

An analysis of the symptoms of gaming disorder, with ANCOVA, showed that when controlling for ADHD, there was no difference between the groups in symptoms of GD according to the GAIT scale [F(1,57) = 1.85, *p* = .18; ADHD F(1,57) = 0.17, *p* = .69]. Furthermore, when comparing the groups regarding the number of DSM criteria in ANCOVA, when controlling for ADHD, it was found that the adults fulfilled, on average. 0.6 criteria more than the AYAs did [F(1,57) = 5.92, *p* = .018; ADHD F(1,57) = 0.95, *p* = .34].

When analyzing gaming days per week, Fisher’s exact test showed no association between gaming days per week and age group (*p* = 0.72). Furthermore, 82.1% of the AYAs and 87.1% of adults reported gaming at least 4 days per week. In addition, Fisher’s exact test showed no association between gaming time on a typical day and age groups (at least 8 h per day, *p* = 0.44; between 6 and 7 h per day, *p* = 0.78; up to 5 h per day, *p* = 0.58). We found that 50% of the AYAs and 38.7% of the adults reported gaming more than 7 h a day on a typical day.

We also assessed the reasons for gaming, and no associations were found between age groups on reasons for gaming according to Fisher’s exact test (escape/coping, *p* = .54; habit, *p* = .61; improve ranking, *p* = 1.0; social, *p* = .63). We also investigated differences in preferred gaming genres. Fisher’s exact test showed a significant association between age group and FPS (*p* = .002), with FPS being more prevalent among the AYAs, and mobile games (*p* = .025) that were more common among the adults. There were no associations between age and any of the other genres (MMORPG, *p* = 1.0; MOBA, *p* = .28; sport games, *p* = .11; other, *p* = 0.49).

#### Clinical measures

Results from the ANCOVAs (controlling for the confounder ADHD regarding PHQ-9, GAD-7, AUDIT and DUDIT) assessing the differences in psychiatric co-morbidity between the younger and the older group with *F*-values and *p*-values are reported in **Table 3**.

**Table 3:** Measures of psychiatric co-morbidity.

Variables		Total (n=69)	Younger/AYA (n=35)	Older/Adult (n=34)	*p*-value	Effect size (Cohen’s *d* / Odds ratio)
**PHQ-9**	Unadjusted	12.2 (5.2)	12.6 (4.6)	11.8 (5.9)		
	Adjusted	12.2 (5.3)	12.9 (4.6)	11.4 (6.1)	0.28	*d* = 0.30
**GAD-7**	Unadjusted	8.3 (5.3)	8.9 (5.9)	7.8 (4.7)		
	Adjusted	8.8 (5.4)	9.0 (6.1)	8.6 (4.6)	0.59	*d* = 0.14
AUDIT *	Unadjusted	4.1 (4.2)	2.9 (2.8)	5.3 (5.0)		
	Adjusted	3.9 (4.0)	2.7 (2.7)	5.3 (4.7)	0.01	*d* = 0.64
**DUDIT**	Unadjusted	1.2 (2.7)	1.1 (2.8)	1.3 (2.7)		
	Adjusted	1.1 (2.5)	1.2 (3.0)	1.0 (2.1)	0.63	*d* = 0.15
**ASRS** (above cut-off) %		56.1	50.0	63.0	0.43	OR = 0.59
**RAADS-14** (above cut-off) %		39.7	43.8	35.5	0.61	OR = 1.41
**NODS-PERC** (above cut-off) %		27.9	37.9	18.8	0.15	OR = 2.65
**Family history of addiction** %		40.0	31.0	48.4	0.20	OR = 0.48
**Tobacco use** %		34.8	27.3	42.4	0.30	OR = 0.51

Data is presented as means and standard deviations *M* (*SD*), in percent (%).

ASRS, RAADS-14, NODS-PERC, Family history of addiction and Tobacco use were calculated with Fisher’s exact test. PHQ-9, GAD-7, AUDIT and DUDIT was calculated with ANCOVA and presented in the table with adjusted and unadjusted means and standard deviations *M* (*SD).*

Effect size is reported as odds ratios for categorical variables and Cohen’s *d* for continuous variables. OR, Odds ratios are reported for AYAs to adults.

*Statistically significant.

AYAs and the older group only differed on the AUDIT scores. It was found that the adults scored higher than the AYAs on the AUDIT scale [F(1,57) = 6.7, *p* = .01; ADHD F(1,57) = 0.10, *p* <.76].

ANCOVA revealed that the two groups did not differ on any of the following measures of psychiatric co-morbidity [PHQ-9 F(1,56) = 1.21, *p* = .28; ADHD F(1,56) = 0.22, *p* = .64; GAD-7 F(1,57) = 0.29, *p* = .59; ADHD F(1,57) = 2.43, *p* = .13; DUDIT F(1,39) = 0.23, *p* = .63, ADHD F(1,39) = 1.62, *p* = .21]. Fisher’s exact test revealed no association between age and ASRS (*p* = .43), RAADS-14 (*p* = .61), NODS-PERC (*p* = .15), family history of addiction (*p* = .20), or tobacco use (*p* = .30).

## Discussion

This cross-sectional study identifies three key findings. First and in line with our hypothesis, we found that the younger treatment-seeking group reported a faster progression into problematic gaming than the adults did. Second and contrary to our hypothesis, we found that both age groups had similar levels of psychiatric symptoms including possible ADHD, ASD, and problematic gambling. Third, notably half of our patient population consisted of adults, 26 years or older.

The faster progression into problematic gaming among the AYAs was one of the most evident differences between the groups in our study. The younger group developed problematic gaming about 7 years after intitiation of gaming while it took 11 years for the adult group to develop problems. Although not significant when controlling for ADHD, the AYAs also reported initiating gaming when they were, on average, 7 years old compared to the adults who began gaming when they were 10 years old. Possible reasons for these differences could be changes in the gaming environment, developmental factors, or a combination thereof. The gaming environment has changed considerably during the last decades. Accordingly, the younger group in our sample have had considerably more access to digital games during childhood and adolescence and access to online games with more addictive potential (13). It is possible that these changes have contributed to the earlier initiation and faster progression to GD in the younger group. Starting gaming at an early age could also in itself be a possible risk factor for developing GD. Our brain undergoes extensive development from childhood to adolescence, making it more susceptible for the development of addiction (21). Previous research has shown that starting gaming at a younger age is associated with GD at older ages (14, 68) and an increased risk of a more severe GD (15). Similar findings have also been reported regarding Internet use (19, 20). This relationship is also well known in the field of substance use disorders. Starting drinking at an early age is associated with an increased risk of faster progression into AUD (23) and a higher risk of ever developing AUD (22). Taken together, this suggests that starting gaming at a younger age may not only increase the risk of developing GD per se but also contribute to a faster progression into GD. From our cross-sectional data, we cannot conclude a causal relationship. Still the observation of a faster progression into GD in the younger group indicates that it could be advisable to be mindful of signs of problematic gaming in early ages as GD might more rapidly develop at that time in life. The continuous changes of the gaming environment also call for further monitoring of how gaming debut and progression into gaming disorder develop as the types of games change and evolve.

Both of our age groups had the same high levels of psychiatric co-morbidity. They reported, on average, a moderate level of depression, and over 50% screened above cut-off for possible ADHD and almost 40% for possible ASD. Symptoms of underlying psychiatric disorders are common in GD, with anxiety, depression, and ADHD being the most prominent (26, 29). Unlike our findings of equal levels of psychiatric symptoms in the younger and older groups, the opposite was seen in a clinical study by Granero et al. (16). They identified an older group of GD patients with higher levels of psychiatric co-morbidity in comparison to a younger group. These differences might be caused by not only differences in methodology but also differences between the samples. The participants in our study reported having had problematic gaming for, on average, 10 years, which is about more than double as long as in the Granero study. It is possible that more psychiatric co-morbidity developed in both of our groups during that amount of time, erasing differences that might have been there at earlier stages. Unfortunately, participants with co-occurring psychiatric symptoms are often excluded in studies investigating treatments for GD (49, 50), which leads to an incomplete picture of this clinical population. More research about psychiatric co-morbidity in representative treatment-seeking samples, covering all age groups, is needed.

Furthermore, substance-related addictions have also been reported in relation to GD, indicating a cross-sensitivity for substance use and behavioral problems (40, 69, 70). In contrast to previous studies, our participants reported a low intake of both alcohol and other substances measured by the AUDIT and DUDIT. Even though we saw that the adult group scored significantly higher on the AUDIT than the AYAs did, the levels were low and several points below the cutoff for problematic use of alcohol (0–7 points) (71). We can only speculate that gaming might have been a protective factor for other addictions in this population, similar to the findings of Erevik et al. (41), or that those with GD in combination with problematic alcohol or substance use seek treatment elsewhere.

Notably, as much as half of our sample consisted of adults. This could seem counterintuitive, as the prevalence of GD is higher among AYAs than in adults (11). One reason for this could be that the age group of adults is larger than the group of AYAs in the general population (72), thus making it possible that an equal or even higher number of adult treatment-seekers could appear even with a lower prevalence rate among adults. This underscores the importance of including older gamers in research. As mentioned in the “Introduction”, only 25% of the treatment studies in five of the most recent systematic reviews and meta-analyses (49–53) included adults over 25 years old. This may lead to an inaccurate representation of the adults in need of treatment for GD. With digital games becoming increasingly available, it is also possible that the age patterns in both prevalence and progression rate into GD can change over time. Irrespectively of age, all our participants had developed GD when seeking treatment. Though the progression rate in young ages might be faster and the risk to develop GD higher, this underscores that GD also can develop after adolescence and in older ages. This makes it necessary to design treatments suitable for adults and make efforts to reach people in need of treatment for GD in all different age groups.

Overall, the preferred genres and reported motives for gaming were similar to the findings in earlier studies. The most common game genres were MMORPGs, FPS, and MOBA games among our participants, genres that often have been reported in combination with GD (12). These types of games often require a heavy investment of time (73), which affects other activities not related to gaming. Previous research has found that spending excessive time playing games like MMORPGs at young ages can impact the development of GD (15, 74). We did, however, see some differences between the age groups, with the younger participants more often preferring FPS while the adults more often played mobile games. Furthermore, a clear majority (80%) reported escapism/coping as an important motive for gaming, which is in line with previous research showing that escapism is strongly associated with GD (45). It has been hypothesized that the association between GD and the escape motive could be understood through the self-medication hypothesis (75) originally suggested in relation to substance use disorders (76). Using gaming as a dominating coping strategy, perhaps to cope with individual vulnerabilities including psychiatric co-morbidities, might be a key factor in maintaining the behavioral addiction (77). This highlights the importance of taking motives for gaming into account in treatment, for example, by offering new emotion regulation strategies when escape is a dominating motive.

Our results should be interpreted with caution. This was an explorative study in a relatively small sample. To start with, we found a near-significant result together with a moderate effect size regarding age differences in gaming debut, indicating that the study could have been underpowered in this aspect. It was further a cross-sectional study, which means that we cannot make any causal conclusions, and the self-report information about such information as age when starting gaming or developing problems could be flawed by, for example, difficulties remembering exact years or periods in one’s life. In addition, the high proportion of male patients in our study sample differs considerably from the gender distribution reported in population studies (11). On the other hand, this mirrors the small number of women in other clinical studies (16, 78, 79) as well as the gender distribution in the total patient population at our clinic. Although this makes the gender distribution skewed, we believe that it is important to include both men and women in studies to accumulate knowledge about who seeks treatment for GD. However, since male patients dominated our patient population, it introduced a bias in the study and therefore limits the generalizability in relation to female gamers. We also had possible ceiling effects in our measurements about the time spent in gaming, which can have obscured possible differences between the groups.

Overall, we used self-reported assessments, which is a common way to collect clinical data in psychological and psychiatric research, yet the method is fallible, and the percentages of different psychiatric conditions are probably higher, after using self-reports, than would be the case after a full diagnostic assessment. Several steps were taken to mitigate these biases. First, we have used well-validated self-report questionnaires, and second, the patients have been able to ask questions about the questionnaires to the clinicians. Third, specifically regarding the GD diagnosis, we have used self-report data in combination with a structured clinical interview to establish a diagnosis as correctly as possible. We have focused on psychiatric co-morbidities but have not included measures of personality traits or personality disorders. Since this study is based on clinical data, the results should first and foremost be interpreted as applying to treatment seekers and not the wider population with GD. The results also need to be confirmed in longitudinal studies.

Increased awareness of GD would be of great importance to both the health sector and the general public. The younger group with a faster progression still reported that it took almost 7 years before they developed problems. This suggests that there is a considerable timeframe where it would be possible to identify at-risk individuals and offer prevention programs before the problems develop into GD. This requires instruments and routines to identify at-risk individuals, preferably in non-medical settings such as schools, to be able to reach them at an early stage. To be able to identify at-risk individuals more effectively, there is also a need to accumulate more knowledge about risk factors such as psychiatric co-morbidities for developing GD at different ages. It would also be valuable with more research about the effects of an early debut of gaming and if interventions that delay gaming onset can reduce the risks of developing GD.

In conclusion we found an association between young age when seeking treatment and a faster progression into GD. This issue is increasingly important as digital games nowadays are available for children of very young ages. At the same time, the large proportion of adults in our clinical sample also underscores the importance of designing treatments for all age groups. From a clinical perspective, the findings that both age groups had similar clinical profiles when seeking treatment indicate that it could be possible to offer treatments with comparable types of interventions to both AYAs and adult patients. With the increasing use of gaming in all age groups, it is timely to be well equipped with both preventive strategies and treatment interventions [e.g., (80)] to counteract the negative effects of excessive gaming.

## Data Availability

The raw data supporting the conclusions of this article will be made available by the authors, without undue reservation.
